# Radial Head Fractures Cluster in the Anterolateral and Anteromedial Quadrants and Do Not Correlate With Coronoid Fracture Types

**DOI:** 10.1097/CORR.0000000000004027

**Published:** 2026-06-26

**Authors:** Nadia Azib, Huub H. de Klerk, Job N. Doornberg, Nadalini Nettuno, Michel P. J. van den Bekerom, Neal C. Chen, Abhiram R. Bhashyam

**Affiliations:** 1 Hand and Arm Research Collaborative, Massachusetts General Hospital, Harvard Medical School, Boston, MA, USA; 2 Department of Orthopaedic Surgery, OLVG, Amsterdam, the Netherlands; 3 Amsterdam Shoulder and Elbow Center of Expertise, OLVG, Amsterdam, the Netherlands; 4 Department of Orthopaedic Surgery, University Medical Centre Groningen and Groningen University, Groningen, the Netherlands; 5 Flinders University, Adelaide, Australia; 6 Faculty of Behavioural and Movement Sciences, Vrije Universiteit Amsterdam, Amsterdam, the Netherlands; 7 Harvard Medical School, Boston, MA, USA

## Abstract

**Background:**

Combined radial head and coronoid fractures, including terrible triad injuries, are challenging patterns associated with elbow instability, poor clinical outcomes, and other complications. While both radial head and coronoid fractures contribute to joint stability, the relationship between their fracture morphologies remains poorly understood. Clarifying these associations may aid in preoperative planning and guide surgical decision-making for restoring elbow stability.

**Questions/purposes:**

(1) In patients with combined coronoid and radial head fractures, what radial head fracture patterns are most common? (2) Are radial head fracture patterns associated with coronoid fracture types?

**Methods:**

This study was a secondary data analysis of a retrospective, multicenter descriptive study of patients with combined coronoid and radial head fractures identified on elbow CT scans. The cohort was derived from two urban tertiary level 1 trauma centers with specialized upper extremity and orthopaedic trauma services. Of 10,016 patients with elbow or forearm injuries who underwent CT imaging, 2% (175) met eligibility criteria for combined coronoid and radial head fractures; eight patients were excluded because of prior pathophysiologic conditions of the elbow, previous surgery, or inadequate imaging quality, leaving 167 patients for analysis, 54% (90) of whom were female, with a mean ± SD age of 50 ± 15 years. The radial head was divided into four quadrants (anterolateral, anteromedial, posterolateral, and posteromedial) using the radial tuberosity as a reference landmark on axial CT images. The anterolateral quadrant corresponds approximately to the surgical “safe zone,” the region opposite the radial tuberosity and in line with the radial styloid. Coronoid fractures were classified according to the O’Driscoll classification, which categorizes fractures into Type 1 (tip fractures involving the coronoid apex), Type 2 (anteromedial facet), and Type 3 (basal fractures involving the coronoid base) patterns. Interrater reliability was determined using the Cohen kappa, yielding substantial agreement for the radial head quadrant involvement (κ = 0.715) and O’Driscoll classification (κ = 0.658) and reflecting typical reliability for CT-based fracture classification, with disagreements resolved through consensus.

**Results:**

The most common radial head fracture pattern was a single fragment involving both the anterolateral and anteromedial quadrants (31% [51 of 167]), followed by two separate fragments involving the anterolateral and anteromedial quadrants (15% [25 of 167]). Most fractures consisted of one fragment (49% [82 of 167]). Radial head fracture patterns were not associated with coronoid fracture type.

**Conclusion:**

Radial head fractures in combined coronoid and radial head fractures most commonly involved the anterolateral and anteromedial quadrants, often as a single fragment. We found no association between coronoid fracture types and radial head fracture patterns, suggesting that these injury components occur independently. These findings may help surgeons anticipate fragment location in relation to surgical exposure, while emphasizing the importance of evaluating the radial head and coronoid as separate elements during preoperative planning and intraoperative assessment.

**Level of Evidence:**

Level IV, prognostic study.

## Introduction

Combined radial head and coronoid fractures, with or without elbow instability attributed to a dislocation (terrible triad injuries), are challenging to treat. These injuries are associated with a higher risk of complications and failure compared with isolated radial head fractures [24]. Radial head fractures are common, accounting for approximately one-third of elbow fractures, and are most frequently classified according to the Mason type classification based on displacement and comminution [12]. Few studies have investigated the association between the number of radial head fracture fragments and coronoid fracture types in combined injuries, and when they do, they rely on the Mason classification [16]. Although these classifications describe fracture severity, they provide limited information regarding the location and orientation of fracture fragments, particularly in the setting of combined injuries. This limitation is relevant for surgical management, as fixation is constrained by limited space available for implant placement, especially when fractures are comminuted. To do so, implants must be positioned subchondrally or within the commonly described “safe zone” of the radial head, located along the anterolateral aspect of the radial head constituting the arc between the radial styloid and the Lister tubercle [2, 25].

Despite the recognized importance of both the radial head and coronoid for elbow stability, the relationship between their fracture morphologies remains poorly understood. Prior studies have largely focused on classifications of the radial head and coronoid process as separate elements, with limited attention to the location and configuration of radial head fragments in combined injuries [3, 16, 19]. In particular, it remains unclear whether specific radial head fracture patterns, such as the number and location of fragments, are associated with distinct coronoid fracture types. Understanding these relationships may help surgeons anticipate fracture morphology and plan surgical exposure and/or fixation strategies in complex elbow injuries [6, 9, 23, 28].

Therefore, we asked: (1) In patients with combined coronoid and radial head fractures, what radial head fracture patterns are most common? (2) Are radial head fracture patterns associated with coronoid fracture types?

## Patients and Methods

### Study Design and Setting

This was a secondary analysis of a previously conducted, retrospective, multicenter diagnostic imaging study designed to characterize fracture morphology in patients with combined coronoid and radial head fractures without an ulna shaft fracture [4]. Patients in the source study were treated at one of two urban level 1 trauma centers (Massachusetts General Hospital, Boston, MA, USA, and the University Medical Center Groningen, the Netherlands), both tertiary referral institutions with specialized upper extremity and orthopaedic trauma services. At these centers, CT imaging is routinely obtained for complex elbow injuries to assess fracture morphology, joint involvement, and surgical planning. This study was conducted in accordance with the Strengthening the Reporting of Observational Studies in Epidemiology (STROBE) guidelines [27]. A detailed description of the original study has been published previously [4], but a brief review follows.

### Patient Selection

For the source study [4], databases were searched with ICD-10 and Current Procedural Terminology (CPT) codes to identify patients with elbow and forearm injuries (ICD-10 S50-S59) who underwent CT scans (CPT 73200-73202, 73206 in the United States; Zorgactiviteitcode [ZA] 084042 in the Netherlands) between February 2014 and March 2023 (Fig. 1). Of 10,016 identified patients (6347 at Massachusetts General Hospital and 3669 at University Medical Center Groningen), 1.7% (175) had concomitant coronoid and radial head fractures on CT and were considered potentially eligible. After excluding 4.6% (8 of 175) of patients for preexisting pathophysiologic conditions of the elbow (n = 6) or poor CT quality (n = 2), 167 patients (95% of eligible) remained for analysis. The majority of patients were excluded during initial screening because of absence of CT imaging, lack of concomitant coronoid and radial head fractures, additional fractures affecting elbow stability, imaging performed outside the study period, or age younger than 18 years. Because CT imaging is typically obtained for more complex injuries, the study population may overrepresent fracture patterns associated with greater instability. Patients were included if they had combined coronoid and radial head or neck fractures, without ulnar shaft or olecranon fractures, and if a CT scan of the elbow was available within 4 weeks of the initial injury, showing no motion artifacts and complete visualization of the elbow’s osseous structure. Patients with prior pathophysiologic conditions of the elbow, surgery, or inconclusive CT findings regarding the presence of fractures were excluded. Fractures were diagnosed on CT scans based on features like cortical step-off, discontinuity, depression, or any displacement or comminution of bone fragments. The review was carried out by two of three researchers (NA, HHK) under the supervision of a fellowship-trained surgeon (ARB), with disagreements resolved through discussion.

**Fig. 1:**
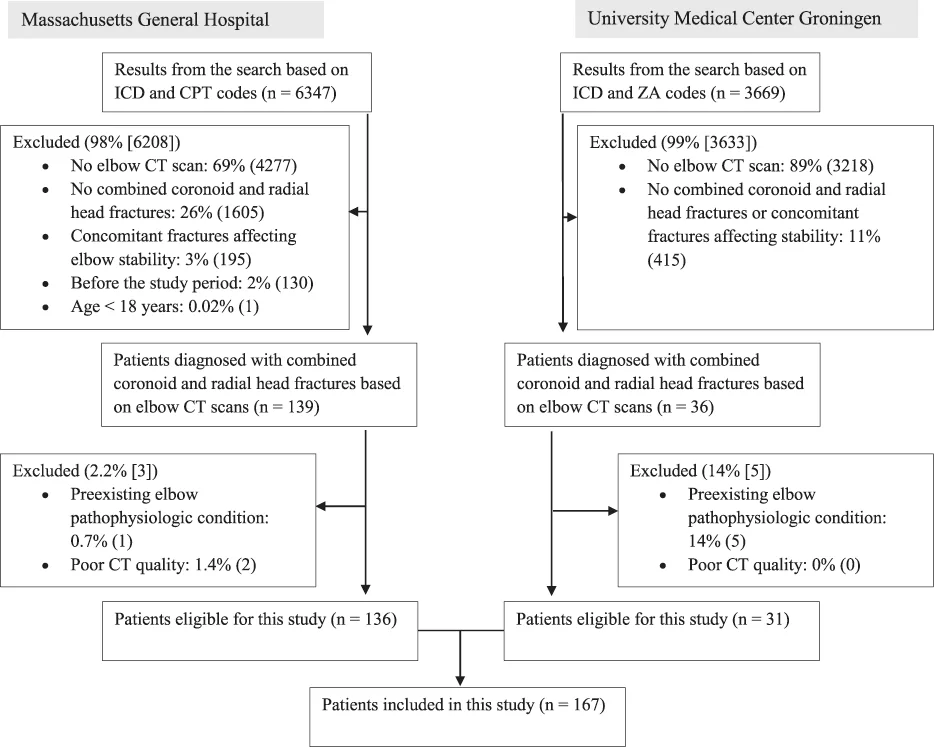
Flow chart of the patient selection process.

### Descriptive Data

The study included 167 patients, with a mean ± SD age of 50 ± 15 years; 54% (90 of 167) were female (Table 1). Posterior dislocation of the ulnohumeral and radiocapitellar joints, characterized by displacement of the ulna from its normal alignment with the humerus and loss of radiographic contact and congruency at both articulations, was observed in 43% (71 of 167) of patients, as identified through plain injury radiographs. Of the 167 patients, 13% (21 of 167) experienced an additional fracture in the affected limb. The most common fractures were humeral lateral epicondyle avulsions and capitellar impactions, each present in 43% (9 of 21) of patients. Other less common fractures included the trochlea, distal radius, and olecranon tip, each accounting for 5% (1 of 21) of the fractures.

**Table 1.** Baseline characteristics of all patients (n = 167)

Characteristic	Female (n = 90)	Male (n = 77)	p value^a^
Age in years	55 ± 14	44 ± 15	< 0.001
Left elbow	57 (51)	53 (41)^b^	0.66
O’Driscoll classification			0.42
Type 1	63 (57)	68 (52)	
Type 2	33 (30)	26 (20)	
Type 3	3 (3)	6 (5)	
Involved radial head quadrants^c^			
Anterolateral	100 (90)	100 (77)	—
Anteromedial	84 (76)	88 (68)	0.47
Posterolateral	57 (51)	52 (40)	0.54
Posteromedial	38 (34)	34 (26)	0.59
Radiocapitellar joint involvement	83 (75)	91 (70)	0.15

Data presented as mean ± SD or % (n).

a Sex-based analysis. Age was compared using an independent-samples t-test; all other variables were compared using Pearson chi-square tests.

b A bilateral fracture was seen in one male patient.

c Multiple quadrants can be affected in one patient.

### Radiographic Assessment

Researchers (NA, HHK), trained by fellowship-trained orthopaedic surgeons (JND, MPJB), independently assessed and classified fractures using two-dimensional and three-dimensional CT scans, followed by confirmation of fracture types through consensus. The evaluation process was standardized to ensure accuracy and reproducibility.

For the secondary analysis, the assessment of the radial head fracture pattern was based on previously published measurements [21, 26]. Displaced fracture fragments of the radial head were identified, and the radial head was divided into quadrants: anterolateral, anteromedial, posterolateral, and posteromedial. To establish a reference point, the most prominent aspect of the radial tuberosity was identified on axial CT images and used as a consistent anatomic landmark to define the posteromedial quadrant, as done by Van Leeuwen et al. [26]. This reference point was then used to determine the orientation of the remaining quadrants, allowing for standardized and reproducible classification across patients. The anterolateral quadrant corresponds to the radial head “safe zone,” defined as the portion of the radial head that is least involved in articulation with the proximal radioulnar joint and therefore permits implant placement with a lower risk of impingement [25]. This region lies approximately opposite the radial tuberosity, traversing the arc between the radial styloid and Lister tubercle (Fig. 2). The involvement of the radial neck was also noted. In patients with associated radial neck fractures, fracture morphology was carefully assessed on CT to determine the relationship between the radial head and shaft. When necessary, the radial head was reoriented to its presumed anatomic position relative to the neck and radial tuberosity by matching fracture lines, allowing consistent quadrant classification despite potential rotational displacement. Each quadrant was assessed independently; patients could have fractures involving multiple quadrants. Therefore, the percentages reported for each quadrant reflect the proportion of the total cohort with involvement in that specific quadrant. Coronoid fractures were classified according to the O’Driscoll classification as Type 1 (tip fractures), Type 2 (anteromedial facet fractures), and Type 3 (basal fractures involving the coronoid base) [22]. The interrater reliability of the radiologic assessment of radial head quadrant involvement was evaluated using the Cohen kappa, with a κ value of 0.715, indicating substantial agreement between raters [15]. The interrater reliability of the radiologic O’Driscoll classification was evaluated using the Cohen kappa, with a κ value of 0.658, indicating substantial agreement between raters [15]. Although these values suggest some variability between observers, they are within the range commonly reported for CT-based fracture classification systems, where moderate to substantial agreement is typical [1, 20]. While the steps we took to standardize assessment may have supported this level of reliability, further improvement would be desirable to enhance consistency and clinical applicability. In instances where the researchers could not reach a consensus on the fracture type, discussions were held with the surgeons to resolve discrepancies and reach a final agreement.

**Fig. 2:**
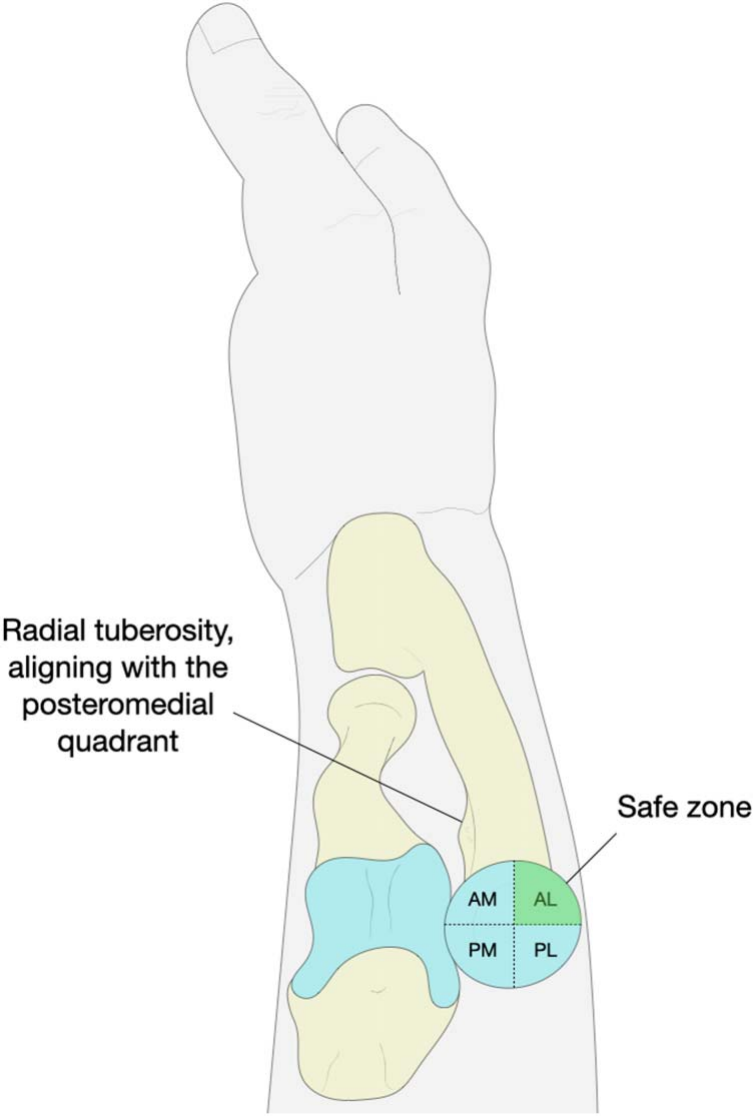
Illustration of the proximal radius and elbow showing the radial head divided into four quadrants: anteromedial (AM), anterolateral (AL), posteromedial (PM), and posterolateral (PL). The radial tuberosity is used as an anatomic landmark aligning with the PM quadrant. The AL quadrant is highlighted as the radial head “safe zone,” the region less involved in proximal radioulnar articulation and therefore commonly considered suitable for implant placement.

### Study Outcomes

Our primary study goal was to assess the fracture patterns of the radial head in combination with coronoid fractures. To achieve this, we analyzed CT scans from patients with combined coronoid and radial head fractures, focusing on the location and type of fractures in each patient. To describe the fracture pattern, we divided the articular part of the radial head into four quadrants based on the radial tuberosity.

Our secondary study goal was to assess whether there is an association between radial head fracture patterns and coronoid fracture types. We classified the coronoid fracture types using the O’Driscoll classification as follows: Type 1 for tip fractures, Type 2 for anteromedial facet fractures, and Type 3 for base fractures. To explore the association, we compared the specific fracture combinations of the radial head and coronoid fractures using chi-square tests.

### Ethical Approval

We obtained ethical approval for this study from the institutional review board at Mass General Brigham (protocol #: 2023P000680) and the METc at University Medical Centre Groningen. The research ethics boards exempted the study from obtaining informed consent.

### Statistical Analysis

Univariate analysis was performed to describe baseline characteristics, including mean ± SDs for continuous variables. Radial head fracture patterns were categorized into three groups based on fragment continuity and configuration: (1) single fragment, defined as a continuous fracture fragment extending across adjacent quadrants; (2) structured two-fragment patterns, defined as symmetric configurations consisting of two distinct fragments; and (3) comminuted complex patterns, defined as all remaining configurations involving multiple fragments that were not structured two-fragment patterns. The association between fracture pattern category and O’Driscoll fracture type was assessed using the Fisher exact test because of small cell counts. The number of fracture fragments was also compared across O’Driscoll fracture types using the Fisher exact test. Data on detailed fracture pattern configurations have been provided and are presented descriptively without separate hypothesis testing (Supplemental Table 1; http://links.lww.com/CORR/B550). The analyses of fracture fragment configuration and number of fragments represent overall comparisons across categories; individual rows are descriptive, and no separate hypothesis tests were performed for each category. Exploratory analyses were performed to assess differences in fracture characteristics by sex. No patients in our cohort identified as nonbinary or gender diverse, and all patients’ reported gender aligned with their biological sex. Age was compared using an independent t-test, and categorical variables—including side of injury, radial head quadrant involvement, and radiocapitellar joint involvement—were analyzed using Pearson chi-square tests. A p value of < 0.05 was considered statistically significant. All statistical analyses were conducted using Stata Statistical Software, release 18 (StataCorp). No formal a priori sample size calculation was performed because this retrospective study included all eligible patients from the participating institutional databases during the study period. At the request of the editors, we performed a post hoc sensitivity power analysis for the main association tests. For the 3 × 3 comparison of radial head fracture pattern category by O’Driscoll coronoid fracture type, a sample size of 167 patients was sufficient to detect an effect size of Cohen w = 0.27 with 80% power at α = 0.05. For the 4 × 3 comparison of the number of radial head fragments by O’Driscoll coronoid fracture type, the minimum detectable effect size was Cohen w = 0.29 under the same assumptions. These values correspond to moderate associations. Power analyses were performed using G*Power, version 3.1 [7, 8].

## Results

### Radial Head Fracture Morphology

The anterolateral and anteromedial quadrants of the radial head were most frequently involved across all O’Driscoll coronoid fracture types (Fig. 3). All fractures involved the anterolateral quadrant (100% [167 of 167]) (Fig. 2). The most common fracture configurations were a single fragment spanning the anterolateral and anteromedial quadrants (31% [51 of 167]) and two separate anterolateral and anteromedial fragments (15% [25 of 167]) (Supplemental Table 1; http://links.lww.com/CORR/B550). Most fractures consisted of one fragment (49% [82 of 167]) (Table 2). We observed no differences between male and female patients in the distribution of quadrant involvement (Table 1).

**Fig. 3:**
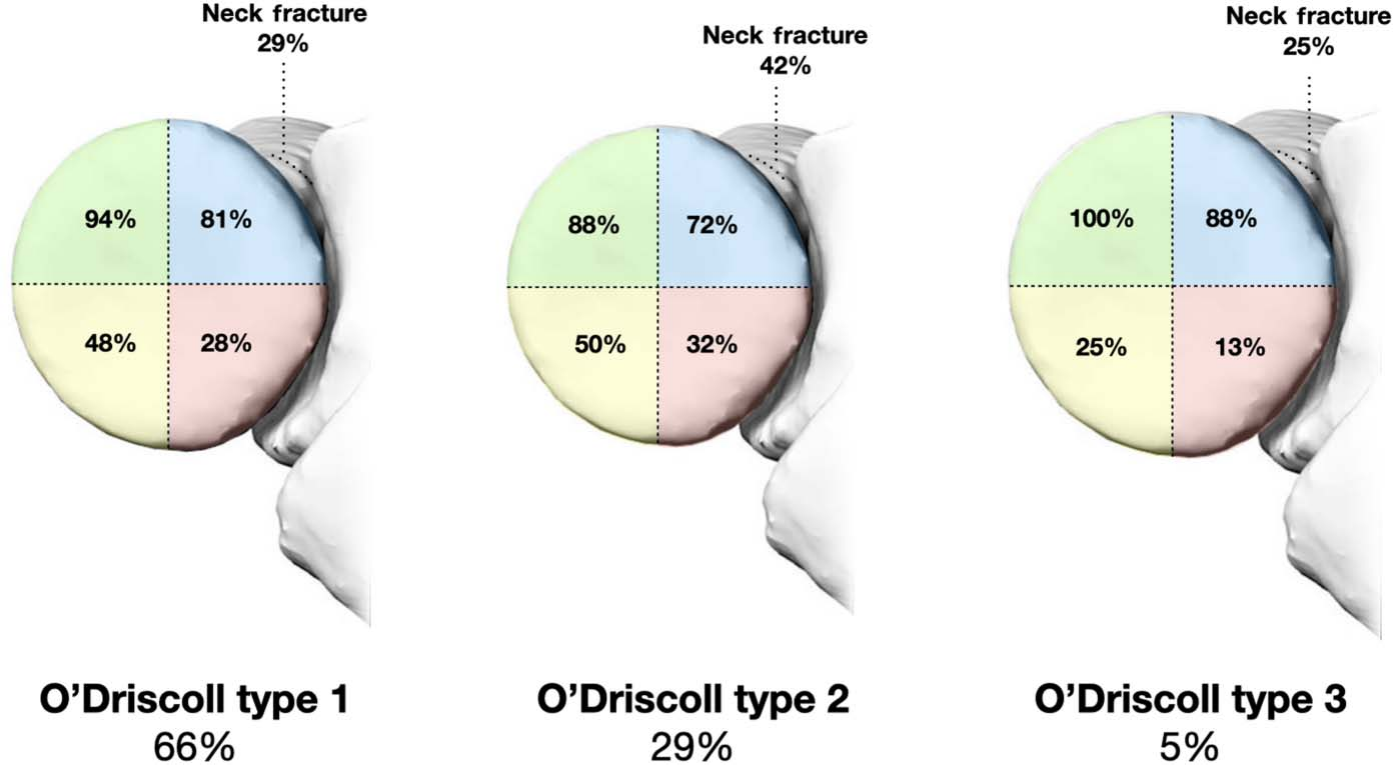
Radial head quadrant involvement per the O’Driscoll coronoid fracture type (green = anterolateral, blue = anteromedial, yellow = posterolateral, red = posteromedial). Each quadrant was assessed independently. Patients may have involvement in multiple quadrants; therefore, percentages are not mutually exclusive and should not be summed.

**Table 2.** Radial head fracture pattern and number of radial head fragments by O’Driscoll classification of coronoid fractures (n = 167)

Variable	O’Driscoll classification of coronoid fractures			
	Type 1 (n = 109)	Type 2 (n = 50)	Type 3 (n = 8)	p value^a^
Radial head fracture pattern^b^				0.93
Single fragment	50 (54)	50 (25)	63 (5)	
Two-fragment (structured)	9.2 (10)	12 (6)	0 (0)	
Comminuted complex	41 (45)	38 (19)	38 (3)	
Number of radial head fragments^c^				0.92
1	48 (52)	50 (25)	63 (5)	
2	19 (21)	20 (10)	0 (0)	
3	13 (14)	10 (5)	13 (1)	
> 3	20 (22)	20 (10)	25 (2)	

Data presented % (n).

a p values were calculated using Fisher exact test.

b Fracture patterns were categorized as single fragment (continuous across quadrants, “+” only), structured two-fragment (symmetric configurations), or comminuted complex (all other multifragment patterns).

c Fragment number reflects the count of discrete fracture fragments (1, 2, 3, > 3).

### Association Between Radial Head and Coronoid Fracture Types

Radial head fracture patterns were heterogeneous and were not associated with coronoid fracture types when grouped into predefined pattern categories (Table 2). A more detailed analysis of individual fracture fragment configurations likewise demonstrated no association across O’Driscoll types (Supplemental Table 1; http://links.lww.com/CORR/B550). There were also no associations between the number of radial head fracture fragments and the coronoid fracture types (Table 2).

## Discussion

Combined coronoid and radial head fractures can jeopardize elbow stability. Given the technical challenges of fixation in these combined injuries, especially because of the limited footprint available for implant placement and the need to avoid impingement, a clear understanding of radial head fracture morphology and potential associations with other fractures is important for preoperative planning. In this study, we found that in injuries with combined coronoid and radial head fractures, the anterolateral and anteromedial quadrants of the radial head were most frequently involved as a single fragment. The anterolateral quadrant corresponds to the radial head safe zone, defined as the portion of the radial head that is nonarticulating with the proximal radioulnar joint and therefore safe for implant placement with a lower risk of impingement. However, fracture extension outside of this region was common, which may influence fixation strategies and implant positioning considerations.

Importantly, radial head fracture morphology was not associated with coronoid fracture type, which suggests that these injury components occur independently rather than in predictable combinations. As such, each fracture component should be evaluated individually during preoperative planning rather than inferred from a presumed associated injury pattern [4]. Because this study was limited to an imaging-based analysis, no conclusions can be drawn regarding fixation methods or clinical outcomes.

### Limitations

This study had several limitations. First, studies have reported that the interobserver and intraobserver reliability for determining radial head fracture morphology using CT scans ranges from poor to fair [11, 18]. Additionally, the reliability of classifying coronoid fractures with the O’Driscoll classification system has been found to be moderate when using both radiography and CT scans [5, 17]. To address this limitation, comprehensive training was provided for our researchers; in addition, we conducted double data collection, and we consulted surgeons in instances of disagreement to ensure accurate fracture-type assessments. Although some degree of misclassification is possible, the use of standardized definitions, independent assessments, and consensus resolution likely limited systematic error. Nonetheless, some variability in classification may remain, and higher reliability is preferable for clinical decision-making. Second, this study may exhibit selection bias as it only included patients who underwent CT scans, potentially skewing the sample toward those with more severe injuries or instability because CT scans are crucial for precise fracture classification [29]. Despite this, most fractures in our cohort consisted of a single fragment and demonstrated consistent involvement of the anterolateral and anteromedial quadrants. This suggests that the observed fracture patterns are likely reflective of injury pattern distribution and not solely attributable to selection bias. Third, although no formal a priori power analysis was performed, this study included all eligible patients available within a limited institutional database over the study period. Because of the nature of the study, increasing the sample size was not feasible, and the findings reflect the complete cohort of patients meeting the inclusion criteria. The post hoc sensitivity power analysis indicated that the sample size was sufficient to detect moderate associations in the main comparisons. Therefore, smaller associations between radial head fracture morphology and O’Driscoll coronoid fracture type cannot be excluded. Nevertheless, any undetected association would likely be modest and would not support using coronoid fracture type alone to infer radial head fracture morphology. In addition, the more detailed fracture configurations included sparse cells and were intended to be descriptive rather than powered for individual pattern-level comparisons. Fourth, rotational displacement of radial neck fractures may influence the perceived orientation of fracture fragments. In our approach, we used CT imaging to evaluate fracture morphology and matched radial head and neck fracture lines to reorient the radial head to its presumed anatomic position relative to the shaft and radial tuberosity. This allowed quadrant classification to be performed with the head and neck aligned, thereby minimizing the effect of rotational displacement. Last, the assessment of instability was based on radiologically confirmed dislocations. This approach excludes patients without radiographic evidence of dislocation, potentially omitting some dislocations that were reduced before imaging, thus creating an information gap. However, relying on patient-reported dislocations was avoided to standardize the injuries utilized for analysis.

### Radial Head Fracture Morphology

Radial head fractures in combined coronoid and radial head injuries most commonly involve the anterolateral and anteromedial quadrants, often as a single large fragment. This consistent distribution may help surgeons anticipate fragment location during preoperative planning, as it relates to their preferred surgical approach and fixation strategy.

Previous clinical and mechanical studies [21, 26] on fracture morphology in (partial) articular fractures of the radial head also identified that the anterolateral quadrant of the radial head was most commonly involved, followed by the anteromedial quadrant [3]. Gordon et al. [10] attributed this to a posterior subluxation to the capitellum and concluded that a shared injury mechanism is responsible for the prevalence of one fracture type over others, a concept supported by Mellema et al. [21]. Additionally, this distribution may be explained by regional variations in bone structure, as the anterolateral portion of the radial head has comparatively less structural support because of thinner articular cartilage and less robust subchondral bone support, which may predispose it to fracture under load [10, 13]. By focusing on combined injuries, our study extends these observations to a more complex injury pattern. Although the anterolateral quadrant corresponds to the radial head safe zone, fracture extension beyond this region was common. In particular, involvement of the posterolateral and posteromedial quadrants was observed in a substantial proportion of cases, underscoring the variability of fracture morphology. These findings indicate that, despite a consistent pattern of anterolateral and anteromedial involvement, fracture configuration should be assessed individually when considering implant placement. Fractures extending beyond the anterolateral and anteromedial quadrants may require advanced fixation strategies, such as specialized plates, countersunk screws, fragment-specific screw fixation, or arthroplasty, to achieve functional recovery [14]. This more structured understanding of fracture morphology may also assist surgeons in anticipating fragment location, selecting between surgical exposures, and performing a systematic intraoperative assessment in complex elbow injuries.

### Association Between Radial Head and Coronoid Fracture Types

There was no consistent, predictable association between radial head fracture morphology and coronoid fracture types. This suggests that coronoid fracture classification does not provide useful information about radial head fracture morphology, and that each fracture component should be evaluated independently. Few studies have investigated the association between the number of radial head fracture fragments and coronoid fracture types in combined coronoid and radial head fractures. A study by Li et al. [16] investigated the association between radial head fracture types according to the Mason classification and coronoid fracture types according to the Regan-Morrey and O’Driscoll classifications in terrible triad injuries. They found that radial head fractures were most often Mason type 2, whereas coronoid fractures were typically Regan-Morrey type 2 and O’Driscoll type 1. No correlation was found in the study by Li et al. [16] for the Regan-Morrey classification with the Mason classification, whereas the O’Driscoll classification had a weak correlation with the Mason classification. Our findings extend this work by using a location-based characterization of radial head fractures and similarly demonstrate the absence of a predictable pattern. Future studies should evaluate whether specific combinations of radial head and coronoid fracture patterns influence surgical decision-making or clinical outcomes, ideally using standardized, location-based classification systems.

### Conclusion

Radial head fractures in combined coronoid and radial head fractures predominantly involve the anterolateral and anteromedial quadrants, often as a single fragment and typically within regions accessible to standard fixation. Still, a considerable number of radial head fractures had additional posterior involvement, which necessitates versatile surgical planning to attain durable fixation because implants placed directly on the radial head in this region would lead to impingement. We found no association between radial head fracture patterns and coronoid fracture types per the O’Driscoll classification, suggesting that these injury components occur independently. These findings may help surgeons anticipate fragment location in relation to the planned surgical exposure, while reinforcing the importance of evaluating the radial head and coronoid as separate elements during preoperative planning and intraoperative assessment. Future studies should aim to simplify fracture mapping for preoperative planning; automatic segmentation and rotational correction of radial head and coronoid fracture fragmentation would make this technique more useful and widely applicable.
